# The Influence of *Hypericum perforatum* L. Addition to Wheat Cookies on Their Antioxidant, Anti-Metabolic Syndrome, and Antimicrobial Properties

**DOI:** 10.3390/foods10061379

**Published:** 2021-06-15

**Authors:** Anna Jakubczyk, Kaja Kiersnowska, Begümhan Ömeroğlu, Urszula Gawlik-Dziki, Krzysztof Tutaj, Kamila Rybczyńska-Tkaczyk, Magdalena Szydłowska-Tutaj, Urszula Złotek, Barbara Baraniak

**Affiliations:** 1 Department of Biochemistry and Food Chemistry, University of Life Sciences in Lublin, Skromna 8, 20-704 Lublin, Poland; anna.jakubczyk@up.lublin.pl (A.J.); urszula.gawlik@up.lublin.pl (U.G.-D.); magdalena.szydlowska@up.lublin.pl (M.S.-T.); barbara.baraniak@up.lublin.pl (B.B.); 2 Scientific Students Group of Food Biochemistry and Nutrition, Department of Biochemistry and Food Chemistry, University of Life Sciences in Lublin, 20-704 Lublin, Poland; kaja.kiersnowska@op.pl; 3 Department of Nutrition and Dietetics, Marmara Üniversitesi Göztepe Yerleşkesi, Kadıköy/İstanbul 34722, Turkey; dyt_begum@hotmail.com; 4 Department of Biochemistry and Toxicology, Faculty of Animal Sciences and Bioeconomy, University of Life Sciences in Lublin, Akademicka 13, 20-950 Lublin, Poland; krzysztof.tutaj@up.lublin.pl; 5 Department of Environmental Microbiology, University of Life Sciences in Lublin, St. Leszczyńskiego 7, 20-069 Lublin, Poland; kamila.rybczynska-tkaczyk@up.lublin.pl

**Keywords:** *Hypericum perforatum* L., antioxidants, metabolic syndrome, cookies

## Abstract

The aim of this study was to characterize wheat cookies enriched with 0.5% and 1.0% of *Hypericum perforatum* L. (St. John’s wort, SJW) and determine their pro-health properties in vitro after hydrolysis in simulated gastrointestinal conditions. The results indicated that 1.0 SJW was characterized by the highest content of polyphenols, flavonoids, and phenolic acids (2.32 mg mL^−1^, 4.93 µg mL^−1^, and 12.35 µg mL^−1^, respectively). The enriching cookies had no effect on water absorption capacity (WAC) and oil absorption capacity (OAC). After in vitro hydrolysis, the highest peptide content was noted in 1.0 SJW (0.52 mg mL^−1^), and the bioactive compounds were characterized by high potential bioaccessibility (PAC), but poor bioavailability (PAV). The addition of SJW increased the ACE, α-amylase, and LOX inhibitory effect, but reduced the inhibition of pancreatic lipase. The highest antioxidant activity was noted for 1.0 SJW. The results showed that only 0.5 SJW and 1.0 SJW had slight antimicrobial activity against *E. coli* ATCC 25922 and *B. cereus* ATCC 14579 with MIC = 12.5 mg mL^−1^. Fractions with molecular mass <3.0 kDa were characterized with the highest p-coumaric acid content. The results show that SJW cookies had a higher content of bioactive compounds and more potent anti-metabolic syndrome effects.

## 1. Introduction

In recent years, there has been a rapid development of the food industry, which has brought some benefits as well as negative effects. Nowadays, the pace of life is frighteningly fast, and people are focused on work and improvement of their career, which results in a lack of time to prepare meals. Consequently, there is a demand for takeaway, ready-to-eat, and pre-prepared meals, which are often processed food [1]. However, it is observed that nutritional decisions have an influence on human health and an increase in the incidence of chronic diseases associated with the development of civilization such as metabolic syndrome, type 2 diabetes, polycystic ovary syndrome (PCOS), tumors, or autoimmune disease [2,3]. Furthermore, obesity is an effect of imbalance between the amount of consumed calories and physical activity [4].

Metabolic syndrome (MS) is a chronic disease comprising insulin resistance, hypertension, obesity, large waist circumference (according to the International Diabetes Foundation: waist circumference of ≥80 cm in women and ≥94 cm in men), and atherogenic dyslipidemia, which are related to each other and associated with higher risk of cardiovascular diseases or type 2 diabetes [5]. It is suspected that hyperglycemia causing insulin resistance is the main factor predisposing to MS, and an essential role is played by chronic inflammation. The development of inflammation is based on changes in metabolic pathways such as mitogen-activated protein kinases (MAPKs), phosphatidyl-inositol 3-kinases (PI3K), nuclear factor kappa-light-chain-enhancer of activated B cells (NF-κB), and c-Jun N-terminal kinase (JNK) [6]. Consequently, insulin resistance leads to an increase in the LDL cholesterol fraction and free fatty acid levels [3]. These changes are involved in the development of atherosclerosis in arteries where pro-inflammatory cytokines (e.g., tumor necrosis factor α (TNF-α) or interleukins) and reactive oxygen species are produced and cause hypertension. Blood pressure disorders are associated with irregularities in the renin–aldosterone–angiotensin system, which is responsible for the regulation of blood pressure and water–electrolyte balance. Angiotensin-1 is formed under the action of the renin enzyme and is then catalyzed to the active form of the hormone angiotensin-2 by an enzyme called angiotensin convertase (ACE), which works by blocking proteins that are designed to dilate blood vessels (e.g., bradykinin). Angiotensin-2 is an important factor in promoting inflammation in the vascular system [7]. Furthermore, the consumption of processed food containing a high amount of sugar and saturated fats is associated with obesity characterized by high levels of free fatty acids (i.e., one of the pro-inflammatory factors) [4]. Besides, it cannot be ignored that obesity and atherosclerosis are correlated with another enzyme involved in immune response—5-lypooxygenase (5-LOX). Its activity is based on producing leukotrienes by catalyzing the transformation of arachidonic acid, which is a substrate in membrane phospholipids. Leukotrienes (LT) are lipids regarded as the strongest inflammatory mediators promoting a variety of chronic diseases [8].

Although consumption of processed food is still high, customer awareness is increasing. Moreover, food processing may convert many ingredients into promoters of inflammation and cause an imbalance of gut microbiome factors. People select good-quality food more often and pay attention to food composition [9,10]. Consequently, producers have been forced to design innovative products that meet customers’ expectations, especially snacks such as cookies and sweets. This type of food is particularly desirable due to its easy availability and is a good basis for designing new products classified as a functional food, which is perceived as a healthier replacement for traditional foodstuffs [11,12]. Enrichment is one of the methods for functional food production. The composition of snacks can be improved using various ingredients (e.g., vitamins, microelements, fiber, or antioxidant substances) contained in numerous species of plants [11]. Bakery products are a good food matrix to obtain products with increased bioactive properties as they are popular with consumers and constitute the main part of their daily diet [13]. 

Research has shown that polyphenols are a common group of bioactive compounds with high antioxidant properties. They are grouped into flavonoid (flavonols, flavanols, flavones, flavanones, isoflavones, and anthocyanins) and non-flavonoid molecules (phenolic acids, hydroxycinnamic acids, lignans, stilbenes, and tannins) [14]. Flavonoids usually occurring in products are categorized as flavonols, flavan-3-ols, flavones, flavanones, anthocyanins, and isoflavones and are characterized by the specific structure C6-C3-C6. Polyphenols are present in fruits, vegetables, nuts, olive, or red wine. They exert strong anti-inflammation, anti-tumor, and anti-atherosclerotic effects, which makes them good factors in the prevention of chronic diseases [15].

*Hypericum perforatum* L., known as St. John’s wort (SJW), has been thoroughly tested and is commonly used in the form of an oil, infusion, or diet supplements [16]. St. John’s wort contains many bioactive compounds that have a positive effect on humans: hypericin (red dye), hyperoside, rutin, quercetin, tannins, and hyperforin [14]. Substances contained in *Hypericum perforatum* are especially known for their influence on mood alteration and anti-depressant effect via synergistic action, especially hypericin and hyperforin [17]. The therapeutic effect of St. John’s wort is obtained through long-term use of substantial amounts of the herb [9]. Moreover, SJW has been described as a plant that may be potentially used in the treatment of many other diseases like AIDS or cancer, and exhibits antioxidant, antidiabetic, or anti-inflammatory properties [18].

The aim of the study was to assess the impact of *Hypericum perforatum* on the antioxidant activity, enzyme inhibitory effect, and antimicrobial properties of wheat flour cookies supplemented with St. John’s wort.

## 2. Materials and Methods

### 2.1. Plant Material

Dry St. John’s wort (*Hypericum perforatum* L.) was purchased at a local market in Istanbul (Turkey).

### 2.2. Procedure for Preparation of Cookies

The cookies were prepared as in Złotek [19] with slight modification: the wheat flour was replaced with the St. John’s wort (SJW) herb at 0.5 and 1 g/100 g (0.5 SJW and 1.0 SJW, respectively). Cookies without St. John’s wort were the control samples (control). All cookies were dried and used for further analysis.

### 2.3. Preparation of Extracts 

The cookies were ground in a laboratory grinder (SM-450C, MRC, Warsaw, Poland). The water extracts were prepared according to the method described by Jakubczyk et al. [20].

### 2.4. Polyphenol Content Assay

#### 2.4.1. Total Phenolic Content (TPC)

Total phenolic content was determined in water extracts and fractions with molecular mass <3.0 kDa using Folin–Ciocalteau reagent [21]. TPC was calculated as gallic acid equivalent (GAE) μg per g of dry weight (DW).

#### 2.4.2. Flavonoid Content Assay (FCA)

FCA was determined with the method described by Lamaison and Carnet [22] and calculated as quercetin equivalent (QE) in μg per g of dry weight (DW).

#### 2.4.3. Phenolic Acid Content Assay

Estimation of total phenolic acids was carried out with the Arnov method [23] and expressed as caffeic acid equivalent (CAE) in μg per g of dry weight (DW).

### 2.5. Reducing Sugar Content 

The reducing sugar content before hydrolysis was determined using the DNS method [24].

### 2.6. Peptide Content Assay

The assay was performed using the Adler–Nissen [25] method with L-leucine as a standard.

### 2.7. Functional Properties

#### 2.7.1. Water Absorption Capacity (WAC)

WAC of the samples was determined according to the method described by Khattab and Arntfield [26] with modification described by Jakubczyk et al. [20].

#### 2.7.2. Oil Absorption Capacity (OAC)

OAC was determined as in Khattab and Arntfield [26] with modification described by Jakubczyk et al. [20].

### 2.8. In Vitro Hydrolysis

In vitro digestion of the cookies was carried out with the method described by Durak et al. [27].

#### Preparation of Fractions with Molecular Mass <3.0 kDa

Fractions with molecular mass <3.0 kDa were obtained according to the method described by Jakubczyk et al. [20].

### 2.9. Potential Bioaccessibility (PAC) and Bioavailability (PAV) of Bioactive Compounds Obtained from Cookies

The theoretical calculation of the nutritional potential was based on the index described by Gawlik-Dziki et al. [28]. The bioaccessibility index (PAC), which is an indicator of the bioaccessibility of bioactive compounds, is expressed as:

PAC = Cph/Cpb
Cph—bioactive compound content in the hydrolyzate
Cpb—bioactive compound content in the sample before hydrolysis

The peptide bioavailability index (PAV), which is an indicator of the bioavailability of bioactive compounds, is expressed as:

PAV = Cpa/Cph
Cpa—bioactive compound content after the absorption process
Cph—bioactive compound content in the hydrolyzate

### 2.10. Antioxidant Activity

#### 2.10.1. ABTS^•+^

Antiradical activity against ABTS^•+^ was determined with the method described by Re et al. [29]. All assays were performed in triplicate. The EC_50_ (mg mL^−1^) value was defined as an effective concentration of the sample that is required to scavenge 50% of radical activity.

#### 2.10.2. DPPH^•^

Antiradical activity against DPPH^•^ was determined with the method proposed by Brand-Williams, Cuvelier, and Berset [30]. All assays were performed in triplicate. The EC_50_ (mg mL^−1^) value was defined as an effective concentration of the sample required to scavenge 50% of radical activity.

#### 2.10.3. Fe^2+^ Chelating Activity

The method developed by Decker and Welch [31] was used to determine the ability of samples to chelate ferrous ion (II). All assays were performed in triplicate.

### 2.11. Determination of Enzyme Inhibitory Activity

#### 2.11.1. Angiotensin Converting Enzyme (ACE) Inhibitory Activity

ACE inhibitory activity was measured by spectrophotometry with o-phtaldialdehyde as in Jakubczyk et al. [32]. The EC_50_ (mg mL^−1^) value, defined as the concentration of the sample that inhibits 50% of ACE activity, was determined by measuring the ACE inhibitory activity and peptide contents of each sample.

#### 2.11.2. Pancreatic Lipase Inhibitory Activity

Lipase inhibitory activity was determined with the method described by Jakubczyk et al. [32].

#### 2.11.3. Lipoxidase (LOX) Inhibitory Activity

The LOX inhibitory assay was carried out with the method described by Szymanowska et al. [33].

#### 2.11.4. α-Amylase Inhibitory Activity

The αGIA was measured with the method described by Jakubczyk, Świeca, Gawlik-Dziki, and Dziki [34].

### 2.12. Antimicrobial Properties

The samples were tested against bacteria *Escherichia coli* ATCC 25922, *Staphylococcus aureus* ATCC 29737, *Listeria monocytogenes* ATCC BBA-2660, *Bacillus cereus* ATCC 14579, and *Salmonella enteritidis* ATCC 4931 as well as yeast *Candida albicans* ATCC 90028. These strains were obtained from the American Type Culture Collection (ATCC, distributors: LGC Standards, Łomianki, Poland) and stored at 4 °C. All strains were cultured at 37 °C on Nutrient Broth (NB) medium. 

#### 2.12.1. Determination of Minimum Inhibitory Concentration (MIC) and Minimum Lethal Concentrations (MLC)

MIC and MLC were determined according to the method described by Jakubczyk et al. [35].

#### 2.12.2. Biotoxicity Assay Using the Resazurin Reduction Method

The biotoxicity against bacteria and yeast was assessed using a resazurine reduction assay as in Jakubczyk et al. [36]. 

### 2.13. Determination of Phenolic Compounds in Fractions with Molecular Mass <3.0 kDa by LC-MS/MS Method

The material (50 mg) was extracted in 13 mL PP test tubes (100 × 16 mm) using 80% methanol. For each sample, 25 μL of internal standard and 10 μg mL^−1^ of 2-thiobarbituric acid (TBA) were added. Samples were extracted by ultrasonication with the use of an Emag Technologies Emmi 30 (Germany) operating at 45 kHz and 200 W for 5 min and shaken for one hour at room temperature. The mixtures were left overnight at 22 °C [37]. Then, the extracts were centrifuged at 12,000× *g* for 20 min and aliquoted in vials for the LC-MS/MS analysis. The concentration of phenolic compounds was determined using a high performance liquid chromatograph (Agilent, Santa Clara, CA, USA) coupled with a mass spectrometer (Ultivo, Agilent). Chromatographic separation was carried out on a Synergi Fusion RP-80 (150 mm × 4.6 mm, 4 µm) column (Phenomenex, Torrance, CA, USA). Mobile phase: A—water with formic acid 0.1% (at the beginning 5%), B—acetonitrile with formic acid 0.1% (95%), 0.5 mL/min flow. Gradient program: 0.0–1.0 min 5% B, 1.0–2.0 min 5–25% B, 2.0–5.0 min 25% B, 5.0–7.0 min 25–30% B, 7.0–13.0 min 30–43% B, 13.0–14.0 min 43–75% B, 14.0–18.0 min 75% B, 18.0–18.5 min 75–95% B, 18.5–20.0 min 95% B, 20.0–20.5 min 95–5% B, 20.5–26.0 min 5% B. The injection volume was 10 µL. For detection, electrospray ionization (ESI) in the negative ion mode was used. Tandem mass spectrometry MS/MS was used for quantitative studies. The parameters of all the molecules monitored with the MRM method (e.g., precursor (Q1), product ions (Q2) as well as collision energy (CE) and retention times) are listed in the Supplementary Materials (Supplementary File Table S1). The LC-MS/MS system was controlled using Agilent MassHunter software, which was also used for data processing.

### 2.14. Statistical Analysis

All determinations were performed in triplicate. Statistical analysis was performed using STATISTICA 13.3. software for mean comparison using ANOVA with Tukey’s post-hoc honestly significant difference (HSD) test at the significance level α = 0.05. 

## 3. Results

### 3.1. Characteristics of Cookie Extracts

In general, the addition of St. John’s wort to the wheat cookies had an impact on the content of phenolic compounds, but had no statistically significant effect on water and oil absorption (Table 1). Sample 1.0 SJW had the highest level of total phenolics, flavonoids, and phenolic acid content (2.32 mg g^−1^, 4.93 mg g^−1^, and 12.95 µg g^−1^, respectively). There were differences between the 0.5 SJW and 1.0 SJW samples in the content of flavonoids and phenolic acids, but the difference between the total phenolic amount was statistically insignificant. It should be noted that the addition of SJW to the cookies had no effect on the WAC and OAC values.

### 3.2. Peptide Content during Hydrolysis

**Figure 1:**
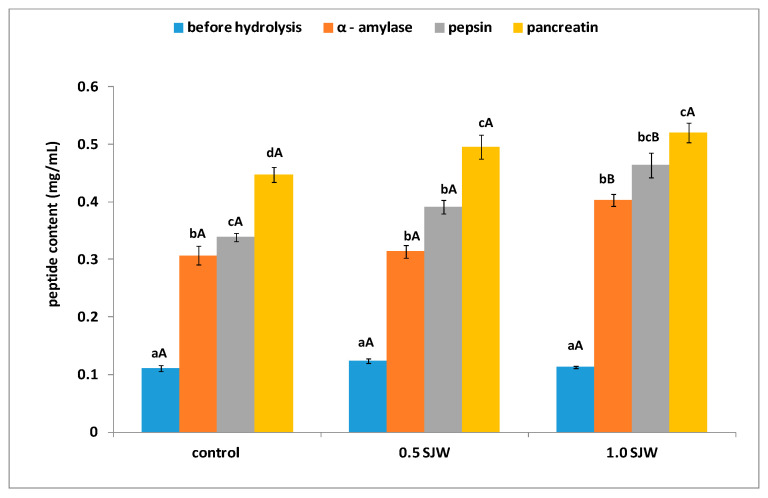
Peptide content during in vitro hydrolysis. Control—cookies without St. John’s wort; 0.5 SJW—cookies enriched with 0.5% of St. John’s wort; 1.0 SJW—cookies enriched with 1% of St. John’s wort. All values are mean ± standard deviation for triplicate experiments. Different capital letters at the same enzyme indicate significant differences for the same indicator (α = 0.05). Different lowercase letters in the same samples indicate significant differences for the same indicator (α = 0.05).

The cookies were subjected to in vitro digestion in gastrointestinal conditions. The peptide content was measured and shown in Figure 1. The α-amylase activity resulted in an increase in the level of peptides in each sample. The 1.0 SJW sample was characterized by the highest level of peptides in each step of hydrolysis compared to the other samples, but only α-amylase and pepsin action exerted a significant effect. The highest peptide content was determined for the 1.0 SJW sample after pancreatin hydrolysis (0.52 mg mL^−1^) but this value was not statistically significant. The differences between the peptide content in the control and the 0.5 SJW sample after α-amylase and pepsin hydrolysis were not statistically significant.

### 3.3. Bioactive Compounds in Hydrolyzates and Fractions with Molecular Mass <3.0 kDa

After hydrolysis, the samples were subjected to the procedure of obtaining fractions with molecular mass <3.0 kDa. Table 2 shows that the polyphenol content was similar in each sample of the hydrolyzates and fractions separately. It should be noted that the fractions contained a statistically significantly higher amount of this bioactive compound. The highest level of flavonoids was observed in the 1.0 SJW sample (29.06 µg g^−1^). The fractions were characterized by a lower content of phenolic acids than the hydrolyzates. In turn, the St. John’s wort had a statistically significant effect addition in the group of hydrolyzates. The same was observed for the values of the peptide content. The content of reducing sugar was statistically the same in the hydrolyzates and fractions.

### 3.4. PAC and PAV Indexes of Bioactive Compounds

Besides the bioactive compound content, we established their potential bioaccessibility and bioavailability factors (Table 3). The compounds exhibited good bioaccessibility in vitro including reducing sugar. The values for all of the samples were higher than 1, and the highest factor for polyphenols and phenolic acids was noted in the 0.5 SJW sample (3.34 and 1.47, respectively) and for flavonoids in the control sample (12.01). The PAC indexes for peptides and reducing sugar were similar (around 4.00 and 0.46, respectively). However, no sample exhibited bioavailability since this factor was lower than one in all cases. The highest PAV index for polyphenols, flavonoids, and reducing sugar was noted for the 0.5 SJW sample (0.83, 0.13, and 0.99, respectively). Moreover, the highest PAV index for phenolic acids and peptides was determined for the control sample (0.29 and 0.72, respectively).

### 3.5. Biological Activity of Hydrolyzates and Fractions with Molecular Mass <3.0 kDa

#### 3.5.1. Antioxidant Activities

The next step consisted in the determination of antioxidant capacities represented by each sample. ABTS·+, DPPH, and Fe^2+^ reagents were used. As shown in Table 4, the addition of St. John’s wort resulted in higher antioxidant activity against ABTS of fractions with molecular mass <3.0 kDa, but it was not statistically significant. Nevertheless, the inactivation capability was lower in the hydrolyzates of the same sample. In the group of fractions, the lowest significant EC_50_ value of the antioxidant activity against DPPH was noted for the control sample = 0.12 mg mL^−1^, but it was not statistically significant compared to the same hydrolyzate sample. The hydrolyzates from the enriched cookies were shown to have an impact on the antioxidant power compared to the control sample. The fractions with molecular mass <3.0 kDa had higher antioxidant properties than the hydrolyzates for the same sample. The Fe^2+^ chelation ability was almost identical for each sample of the hydrolyzates and fractions. The highest antioxidant activity was observed for the control and 0.5 SJW samples (87.23% and 87.09%, respectively), but these values were not statistically significant compared to the rest of the results.

#### 3.5.2. Assay of Inhibition of Enzymes Involved in Metabolic Syndrome Pathogenesis

The impact of the hydrolyzates and fractions with molecular mass <3.0 kDa on enzymes involved in metabolic syndrome pathogenesis was investigated. Clearly, the highest influence on enzyme inhibition was exhibited by the fractions, especially in the case of α-amylase. As shown by the data in Table 5, the 1.0 SJW hydrolyzate samples from cookies enriched with St. John’s wort exhibited increased activity against ACE, LOX, and α- amylase function (EC_50_ 1.24 mg mL^−1^, 1.03 mg mL^−1^, and 0.05 mg mL^−1^, respectively), but the value of ACE inhibition was not statistically significant. The lowest effect of the St. John’s wort addition was observed for lipase activity.

### 3.6. Antimicrobial Effect

**Figure 2:**
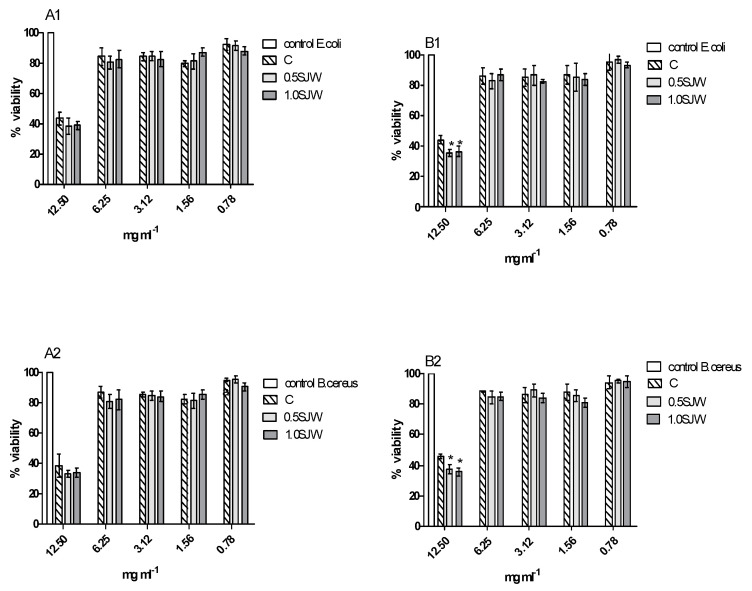
Viability (in %) of *E. coli* ATCC 25922 and *B. cereus* ATCC 14579 against hydrolyzates (**A1**,**A2**) and fractions (**B1**,**B2**) of samples: 0.5 SJW, 1.0 SJW, and control (cookies without St. John’s wort).

The antimicrobial properties of the samples were tested against bacteria *Escherichia coli* ATCC 25922, *Staphylococcus aureus* ATCC 29737, *Listeria monocytogenes* ATCC BBA-2660, *Bacillus cereus* ATCC 14579, and *Salmonella enteritidis* ATCC 4931 and yeast *Candida albicans* ATCC 90028. The results showed that only 0.5 SJW and 1.0 SJW had slight antimicrobial activity against *E. coli* ATCC 25922 and *B. cereus* ATCC 14579 with MIC = 12.5 mg mL^−1^ (Supplementary File Table S2). The results were confirmed by the resazurin assay. It showed that in the presence of the 0.5 SJW and 1.0 SJW samples, *E. coli* ATCC 25922 and *B. cereus* ATCC 14579 were characterized by a similar resazurin reduction degree. The viability of *E. coli* ATCC 25922 and *B. cereus* ATCC 14579 was inhibited by 40–60% and 50–70% at the concentration of 12.5 mg mL^−1^, respectively (Figure 2). Fractions with molecular mass <3.0 kDa from 0.5 SJW and 1.0 SJW inhibited the growth of *E. coli* and *B. cereus* slightly, but statistically significantly (α = 0.05) compared to the control sample (cookies without St. John’s wort).

### 3.7. Phenolic Compounds in Fractions with Molecular Mass <3.0 kDa Identified with the LC-MS/MS Method

Phenolic compounds identified by the LC-MS/MS method are shown in Table 6. It should be noted that not all standards were identified in the tested samples. The addition of St. John’s wort exerted an effect on the content of most of the identified compounds. The highest concentration was noted for p-coumaric acid in the 1.0 SJW sample, but gallic acid and salicylic acid were determined under a lower limit of quantitation (LLOQ = 0.5 µg g^−1^).

## 4. Discussion

For the last few years, food has been considered by consumers not only as a source of nutritional compounds, but also as a source of bioactive compounds that may provide various benefits to human health. Due to the pandemic situation of SARS-CoV-2 and COVID-19 disease, people spend more time at home, have low physical activity, and undergo more stress. For this reason, more attention should be paid to nutrition, which should contain compounds with antioxidant activity, preventing the occurrence of diseases such as hypertension, obesity, or diabetes 2.

Food enrichment is one of the methods of increasing the pro-health quality and compound quality of products. This process is applied to many types of food products. The most commonly enriched food is bread [38,39,40], pasta [41,42,43] or snacks such as wafers [20], crackers [44], and cookies [19]. Food enrichment changes the texture, flavor, or aroma of products and alters the technological properties (e.g., baking value or storage conditions). Moreover, it may cause the appearance of new features that were not present in the product before. The additives represent various types of ingredients: waste products, flour, oil, fruit, vegetables, and herbs.

Herbs not only improve the taste and aroma of products but may also have several health properties (e.g., antioxidant, anticancer, anti-inflammatory, or antimicrobial activity) and are applied in the production of many cereal products. The aim of our study was to investigate the influence of *Hypericum perforatum* L., known as St. John’s wort (SJW), on the bioactive compounds and biological activity of wheat cookies. This herb is characterized by a high content of bioactive compounds (e.g., polyphenols, flavonoids, biflavones, phenolic acids, and naphthodianthrones). The plant is used as an alternative method primarily in the treatment of depression as well as rheumatism, gastroenteritis, headache, and neuralgias. The exact mechanism of action is still unknown, but there are studies that have indicated the role of the neurotransmitter inhibitory profile and potential anti-inflammatory and antioxidant effects, suggesting its role in pain treatment [45].

The research material was three types of cookies: control—without the addition of SJW; 0.5 SJW—cookies with 0.5 of St. John’s wort; and 1.0 SJW—cookies with 1% of St. John’s wort. Since SJW is characterized by high polyphenol content, the total polyphenols, flavonoids, and phenolic acids in extracts obtained from the cookies were determined (Table 1). As expected, the addition of SJW to the cookies increased the levels of bioactive compounds. It should be noted that the total phenolic content in 0.5 SJW and 1.0 SJW was higher than that in the control sample, but the difference between 0.5 SJW and 1.0 SJW was not statistically significant. The present results are in agreement with the findings reported by Złotek [19], where the total phenolic content in cakes enriched with 1% and 2% of basil was higher than in the control sample, but the difference between C1% and C2% was not statistically significant. In turn, the content of flavonoid and phenolic acid was not statistically significantly higher in the control and 0.5 SJW samples. The addition of St. John’s wort increased the content of these bioactive compounds only in the case of 1.0 SJW. This indicates that the food matrix has an influence on the extraction of bioactive compounds [46]. It should be noted that the enriched cookies did not have different WAC and OAC values to the control sample. WAC is an economically important factor for the food industry as loss of moisture adversely affects the yield and quality of products [47]. Hence, some products, especially meat, should be characterized by a high WAC value. However, this factor for cereal products should not be as high, and water absorption levels are often in the range of 60–62% in the standard white bread formula, 80–90% in the artisan-type Ciabatta formula, and 50–54% in the cookie formula [48]. Wheat flour is characterized by 75% WAC [49]. As shown in Table 1, the WAC value of all samples was around 100%, and this is not a high value compared with products enriched with other additives. In our previous research, wheat wafers were enriched with millet flour and the WAC value was above 600% [20]. Another important functional property of cereal products is the absorption capacity (OAC), which contributes to enhancing mouth feel while retaining the flavor of the food product. The ability of food ingredients to bind with oil makes them useful in food production with optimal oil absorption. OAC facilitates enhancement of the flavor and mouth feel when used in food preparation [48]. In our study, the addition of St. John’s wort to the cookies had no statistical effect on the increase in this parameter. All samples were characterized by around 160% OAC (Table 1), which indicates that this factor may influence flavor formulation.

All samples were hydrolyzed in vitro in gastrointestinal condition and, afterward, fractions with molecular mass <3.0 kDa were obtained from the hydrolyzates. This fraction potentially contains compounds that can pass through the lumen of the small intestine [50]. In each step of the hydrolysis, the content of peptides was determined (Figure 1). During the hydrolysis, α-amylase was used as the first active enzyme in the gastrointestinal tract. This enzyme hydrolyzes carbohydrates, but the results of our study indicated increased peptide content in the sample after action of this enzyme. This process was also observed in our previous study [20,51,52], which indicated that proteins are combined with polysaccharides and may release protein or peptides when exposed to this enzyme. The results of the present study demonstrated that the addition of SJW to the cookies induced no significant differences in the peptide content, which was noted only between 0.5 SJW and 1.0 SJW after the use of α-amylase and pepsin. The highest peptide content was noted for hydrolyzates obtained from 1.0 SJW after pancreatic hydrolysis (0.52 mg mL^−1^), but the values after the last step of hydrolysis of all samples were not statistically significant. 

The content of bioactive compounds was determined in all hydrolyzates and fractions with molecular mass <3.0 kDa. The results show (Table 2) that all hydrolyzates were characterized by a higher content of bioactive compounds than the fractions. Statistical significance was noted for flavonoids in the 1.0 SJW sample and phenolic acids and peptides in the 0.5 SJW and 1.0 SJW samples. These results indicate that the addition of St. John’s wort to cookies had no significant influence on the release of bioactive compounds from the food matrix. Similar results were obtained from the fraction where only reducing sugar content was different in 1.0 SJW from that in the other samples. Besides the content of bioactive compounds in the food matrix, their potential bioaccessibility (PAC) and bioavailability (PAV) factors are very important. All PAC factors were significantly higher than 1, which indicated high potential bioaccessibility of the bioactive compounds (Table 3). These results correspond well with those described by Świeca et al. [53], where the PAC index for wheat bread enriched with green coffee was also higher than 1. The PAC indexes for all bioactive compounds (except for flavonoids) in the enriched cookies were higher than in the control sample. This suggests that the addition of St. John’s wort to cookies may indicate the occurrence of phenolic–cookie matrix interactions. This process was also described for enriched bread [54]. On the other hand, the study results showed that the bioactive compounds obtained from cookies enriched with St. John’s wort were characterized by low potential bioavailability. For all compounds, the PAV index was lower than 1, but the 0.5% addition of herbs induced an increase in the potential bioavailability of polyphenols, flavonoids, and reducing sugar (Table 3).

St. John’s wort has been recognized as a source of bioactive compounds with antioxidant, antidepressant, anti-apoptotic, anti-obesity, antimicrobial, antiviral, antioxidant, anti-inflammatory, and enzyme inhibitory effects [55]. The exact mechanism of action is still not understood. Nevertheless, St. John’s wort components have been described to exert inhibitory on hydrolases such as acetylcholinesterase, α-glucosidase, and α-amylase [56]. For this reason, St. John’s wort was chosen as an additive to the formula of cookies. As shown by the results (Table 4), the fractions with molecular mass <3.0 kDa were characterized by higher antioxidant effects than the hydrolyzates. The strongest antiradical effect was noted for sample 1.0 SJW, which indicates that enrichment of cookies with St. John’s wort is an effective method to obtain a product that may be used for its therapeutic diet effect. Excessive concentrations of free radicals and weakened defense mechanisms against them can be the cause of many diseases (e.g., cardiovascular diseases, obesity, diabetes, and inflammation processes). These disorders are also a risk factor of the development of metabolic syndrome, which is considered as the leading cause of death [57]. It is believed that the disease will be even more prevalent due to the epidemic situation. An incorrect lifestyle including inappropriate diet, low physical activity, or stress plays a key role in the development of this disease. At the molecular basis, there is the activity of some enzymes, especially hydrolases such as the angiotensin converting enzyme (ACE), which may cause hypertension and cardiovascular diseases, pancreatic lipase that hydrolyzes about 70% of fat from food products in the human gastrointestinal tract, and amylases that hydrolyze polysaccharides into small molecules. Moreover, the inflammation process may generate free radicals and may contribute to the development of obesity. One of the therapeutic methods for reducing the development of metabolic syndrome is the inhibition of the activity of some enzymes. In the present study, we investigated the inhibitory effects of hydrolyzates and fractions with molecular mass <3.0 kDa on their activity. As shown by the present results (Table 5), the addition of St. John’s wort increased the inhibitory effect of the product. The highest anti-metabolic effect was noted for the enriched samples. Only the lipase inhibitory activity was not stronger than that of the control sample. The hydrolyzates were characterized by a significantly lower effect than the fractions with molecular mass <3.0 kDa, which may indicate potential bioaccessibility. It should be noted that the reducing sugar content, which may contribute to an increase in postprandial glycemia, was the same in the hydrolyzates and the fractions. In turn, the α-amylase inhibitory effect of the fraction was significantly higher than that of the hydrolyzates. This suggests that the obtained products can be used for a therapeutic diet effect in diabetes.

Foodborne pathogens are responsible for foodborne infections with significant effects on human health. Most food poisoning reports are associated with bacterial contamination, especially by Gram-negative bacteria *Escherichia coli* and Gram-positive bacteria *Bacillus cereus* [58]. Consequently, there is a growing interest in functional food ingredients of natural origin, which may affect health in addition to their nutritional value. Our study showed slight but significant antimicrobial activity of cookies enriched with 12.5 mg mL^−1^ of St. John’s wort against *E. coli* and *B. cereus*. Avato et al. [59] reported that St. John’s wort ethanolic extract inhibited only the growth of Gram-positive bacteria *B. subtilis* and *B. cereus.* The authors indicated hypercin and hyperforin as the main antibacterial agents [59]. However, other authors detected antimicrobial activity of *H. perforatum* not only against *E. coli* and *B. cereus*, but also against *Shigella dysenteriae*, *Salmonella typhi*, *Staphylococcus aureus*, *Streptococcus mutans*, and *Enterococcus faecalis* [60,61,62].

The identification by the LC-MS/MS method determined that the addition of St. John’s wort into wheat cookies preparation had an influence on the content of phenolic compound in fraction with a molecular mass <3.0 kDa. As shown by the data in Table 6, phenolic compounds in samples were gallic acid, chlorogenic acid, 3,4-dihydroxybenzoic acid, rutin, p-coumaric acid, ferulic acid, and salicylic acid. The results corresponded well with that described by Silwa et al. [63] where these compounds were also determined. The highest content of phenolic compounds was noted in the 1.0 SJW sample. It should be noted that the addition of 1.0% of St. John’s wort decreased potential bioaccessibility of bioactive compounds such as phenols.

## 5. Conclusions

Food enrichment is one of the effective methods of increasing the pro-health potential of products. This process is not only aimed at increasing the pro-health value of food, but can also influence the taste, smell, and texture. The study indicated that cookies enriched with St. John’s wort had a higher content of bioactive compounds and antioxidant and anti-metabolic syndrome effects. These results showed that *Hypericum perforatum* L. has good potential to be used for the production of potential functional food with not only anti-depression properties.

## Figures and Tables

**Table 1:** Phenolic compound content and functional properties of extracts obtained from cookies.

Sample	Polyphenol Compound Content			Functional Properties	
	Total Phenolic Content (mg gDW)	Flavonoids (µg gDW)	Phenolic Acids (µg gDW)	WAC (%)	OAC (%)
Control	1.97 ± 0.06 ^a^	2.11 ± 0.30 ^a^	7.65 ± 0.86 ^a^	91.45 ± 14.18 ^a^	162.76 ± 1.59 ^a^
0.5 SJW	2.11 ± 0.18 ^b^	2.29 ± 0.27 ^a^	8.95 ± 1.08 ^a^	117.26 ± 10.64 ^a^	161.50 ± 4.86 ^a^
1.0 SJW	2.32 ± 0.05 ^b^	4.93 ± 0.47 ^b^	12.35 ± 0.68 ^b^	101.70 ± 5.54 ^a^	161.26 ± 2.75 ^a^

Control—cookies without St. John’s wort; 0.5 SJW—cookies enriched with 0.5% of St. John’s wort; 1.0 SJW—cookies enriched with 1% of St. John’s wort. All values are mean ± standard deviation for triplicate experiments. All values are mean ± standard deviation for triplicate experiments. Different letters indicate significant differences (α = 0.05).

**Table 2:** Bioactive compounds in hydrolyzates and fractions with molecular mass <3.0 kDa.

Samples	Polyphenols (mg gDW)	Flavonoids (µg gDW)	Phenolic Acids (µg gDW)	Peptides (mg gDW)	Reducing Sugar (mg gDW)
	Hydrolyzates				
Control	4.77 ± 0.10 ^aA^	25.38 ± 1.28 ^aA^	9.03 ± 1.43 ^aA^	8.91 ± 0.26 ^aA^	8.10 ± 0.30 ^aA^
0.5 SJW	4.94 ± 0.17 ^aA^	26.29 ± 1.02 ^aA^	13.17 ± 1.27 ^bB^	10.41 ± 1.12 ^bB^	8.03 ± 0.43 ^aA^
1.0 SJW	5.11 ± 0.18 ^aA^	29.06 ± 0.42 ^bB^	14.59 ± 0.46 ^bB^	11.26 ± 0.34 ^bB^	8.50 ± 0.44 ^aA^
Fractions with molecular mass <3.0 kDa					
Control	3.90 ± 0.19 ^aB^	3.09 ± 0.17 ^aC^	2.58 ± 0.07 ^aC^	6.48 ± 0.38 ^aC^	8.09 ± 0.57 ^aA^
0.5 SJW	4.08 ± 0.28 ^aB^	3.32 ± 0.52 ^aC^	2.66 ± 0.36 ^aC^	6.94 ± 0.49 ^aC^	8.01 ± 1.07 ^aA^
1.0 SJW	3.98 ± 0.28 ^aB^	3.76 ± 0.83 ^aC^	2.83 ± 0.52 ^aC^	7.04 ± 0.52 ^aC^	8.28 ± 0.87 ^aA^

Control—cookies without St. John’s wort; 0.5 SJW—cookies enriched with 0.5% of St. John’s wort; 1.0 SJW—cookies enriched with 1% of St. John’s wort. All values are mean ± standard deviation for triplicate experiments. Different capital letters in the same column indicate significant differences for the same indicator (α = 0.05). Different lowercase letters in the same type of samples indicate significant differences for the same indicator (α = 0.05).

**Table 3:** PAC and PAV indexes of the bioactive compounds from cookies.

Samples	Polyphenols	Flavonoids	Phenolic Acids	Peptides	Reducing Sugar
PAC					
Control	2.42	12.01	1.12	4.04	0.46
0.5 SJW	3.34	11.50	1.47	4.00	0.46
1.0 SJW	2.20	7.43	1.18	4.60	0.49
PAV					
Control	0.81	0.12	0.29	0.72	0.70
0.5 SJW	0.83	0.13	0.20	0.65	0.99
1.0 SJW	0.77	0.10	0.19	0.62	0.97

Control—cookies without St. John’s wort; 0.5 SJW—cookies enriched with 0.5% of St. John’s wort; 1.0 SJW—cookies enriched with 1% of St. John’s wort. All values are mean ± standard deviation for triplicate experiments.

**Table 4:** Antioxidant properties of hydrolyzates and fractions with molecular mass <3.0 kDa.

Samples	ABTS (EC_50_ mg mL^−1^)	DPPH (EC_50_ mg mL^−1^)	Fe^2+^ Chelation (%)
Hydrolyzates			
Control	0.92 ± 0.07 ^aA^	1.37 ± 0.07 ^bD^	84.20 ± 2.18 ^abAB^
0.5 SJW	5.32 ± 0.18 ^bC^	0.99 ± 0.04 ^aC^	82.96 ± 2.93 ^aA^
1.0 SJW	4.38 ± 0.38 ^cB^	1.06 ± 0.1 ^aC^	86.98 ± 2.50 ^bB^
Fractions with molecular mass <3.0 kDa			
Control	0.76 ± 0.02 ^bA^	0.60 ± 0.05 ^cB^	87.23 ± 0.67 ^aB^
0.5 SJW	0.54 ± 0.05 ^aA^	0.51 ± 0.02 ^bB^	87.09 ± 1.67 ^aB^
1.0 SJW	0.53 ± 0.01 ^aA^	0.12 ± 0.002 ^aA^	86.39 ± 1.32 ^aAB^

Control—cookies without St. John’s wort; 0.5 SJW—cookies enriched with 0.5% of St. John’s wort; 1.0 SJW—cookies enriched with 1% of St. John’s wort. All values are mean ± standard deviation for triplicate experiments. Different capital letters in the same column indicate significant differences for the same indicator (α = 0.05). Different lowercase letters in the same type of samples indicate significant differences for the same indicator (α = 0.05).

**Table 5:** Hydrolyzates and fractions with molecular mass <3.0 kDa as inhibitors of enzymes involved in metabolic syndrome (EC_50_ mg mL^−1^).

	Enzyme	ACE	Lipase	LOX	α-Amylase
Samples					
Hydrolyzates					
Control		1.52 ± 0.29 ^aA^	0.57 ± 0.02 ^aA^	2.33 ± 0.22 ^bC^	3.77 ± 0.06 ^aC^
0.5 SJW		1.26 ± 0.09 ^aA^	1.48 ± 0.05 ^bB^	2.47 ± 0.56 ^bC^	1.72 ± 0.06 ^bB^
1.0 SJW		1.24 ± 0.03 ^aA^	1.90 ± 0.15 ^cB^	1.03 ± 0.02 ^aB^	0.05 ± 0.008 ^cA^
Fractions with molecular mass <3.0 kDa					
Control		0.46 ± 0.03 ^aB^	0.12 ± 0.005 ^aA^	0.44 ± 0.4 ^aAB^	0.029 ± 0.006 ^bA^
0.5 SJW		0.33 ± 0.04 ^bB^	0.17 ± 0.005 ^abA^	0.24 ± 0.001 ^bA^	0.019 ± 0.006 ^abA^
1.0 SJW		0.58 ± 0.05 ^cB^	0.19 ± 0.04 ^bA^	0.076 ± 0.001 ^cA^	0.013 ± 0.003 ^aA^

Control—cookies without St. John’s wort; 0.5 SJW—cookies enriched with 0.5% of St. John’s wort; 1.0 SJW—cookies enriched with 1% of St. John’s wort. All values are mean ± standard deviation for triplicate experiments. Different capital letters in the same column indicate significant differences for the same indicator (α = 0.05). Different lowercase letters in the same type of samples indicate significant differences for the same indicator (α = 0.05).

**Table 6:** Composition of phenolic compounds identified in fractions µg g^−1^ DW.

Phenolic Compounds	Sample		
	Control	0.5 SJW	1.0 SJW
gallic acid	<0.5	<0.5	<0.5
chlorogenic acid	nd	nd	1.08
3,4-dihydroxybenzoic acid	nd	2.28	3.61
rutin	nd	6.07	11.56
p-coumaric acid	9.72	15.48	23.88
ferulic acid	2.10	4.01	4.64
salicylic acid	<0.5	<0.5	<0.5

DW—dry weight; nd—not detected.

## Data Availability

All relevant data are included in the article.

## Supplementary Materials

The following are available online at https://www.mdpi.com/article/10.3390/foods10061379/s1, Table S1. The parameters for all the molecules monitored by MRM method: precursor (Q1), product ions (Q2) as well as collision energy (CE) and retention times, Table S2. Antimicrobial activity of tested samples towards bacteria and yeast; MIC—minimum inhibitory concentration, MLC—minimum lethal concentrations, ns—not studied.

## References

[1] Guiné R.P.F., Florença S.G., Barroca M.J., Anjos O. (2020). The link between the consumer and the innovations in food product development. Foods.

[2] Bagheri M., Willett W., Townsend M.K., Kraft P., Ivey K.L., Rimm E.B., Wilson K.M., Costenbader K.H., Karlson E.W., Poole E.M. (2020). A lipid-related metabolomic pattern of diet quality. Am. J. Clin. Nutr..

[3] Barber T.M., Kyrou I., Randeva H.S., Weickert M.O. (2021). Mechanisms of insulin resistance at the crossroad of obesity with associated metabolic abnormalities and cognitive dysfunction. Int. J. Mol. Sci..

[4] Ravaut G., Légiot A., Bergeron K.F., Mounier C. (2021). Monounsaturated fatty acids in obesity-related inflammation. Int. J. Mol. Sci..

[5] Dandona P., Aljada A., Chaudhuri A. (2005). Metabolic Syndrome A Comprehensive Perspective Based on Interactions between Obesity, Diabetes, and Inflammation. Circulation.

[6] Novelli M., Masiello P., Beffy P., Menegazzi M. (2020). Protective role of st. John’s wort and its components hyperforin and hypericin against diabetes through inhibition of inflammatory signaling: Evidence from in vitro and in vivo studies. Int. J. Mol. Sci..

[7] Chen J., Ryu B., Zhang Y., Liang P., Li C., Zhou C., Yang P., Hong P., Qian Z. (2020). Comparison of an angiotensin-I-converting enzyme inhibitory peptide from tilapia (*Oreochromis niloticus*) with captopril: Inhibition kinetics, in vivo effect, simulated gastrointestinal digestion and a molecular docking study. J. Sci. Food Agric..

[8] He Z., Tao D., Xiong J., Lou F., Zhang J., Chen J., Dai W., Sun J., Wang Y. (2020). Phosphorylation of 5-LOX: The Potential Set-point of Inflammation. Neurochem. Res..

[9] Lawvere S., Mahoney M. (2005). St. John’s Wort. Am. Fam. Physician.

[10] Petrescu D.C., Vermeir I. (2020). Consumer Understanding of Food Quality, Healthiness, and Environmental Impact: A Cross-National Perspective. Int. J. Environ. Res. Public Health.

[11] Antonic B., Dordevic D., Jancikova S., Holeckova D., Tremlova B., Kulawik P. (2021). Effect of Grape Seed Flour on the Antioxidant Profile, Textural and Sensory Properties of Waffles. Processes.

[12] Laster J., Frame L.A. (2019). Beyond the Calories—Is the Problem in the Processing?. Curr. Treat. Options Gastroenterol..

[13] Longoria-García S., Cruz-Hernández M.A., Flores-Verástegui M.I.M., Contreras-Esquivel J.C., Montañez-Sáenz J.C., Belmares-Cerda R.E. (2018). Potential functional bakery products as delivery systems for prebiotics and probiotics health enhancers. J. Food Sci. Technol..

[14] Lorenzo C.D., Colombo F., Biella S., Stockley C., Restani P. (2021). Polyphenols and Human Health: The Role of Bioavailability. Nutrients.

[15] Li Y., He D., Li B., Lund M.N., Xing Y., Wang Y., Li F., Cao X., Liu Y., Chen X. (2021). Polyphenols with biological functions via polyphenol-protein interactions as additives for functional foods. Trends Food Sci. Technol..

[16] Schepetkin I.A., Ozek G., Ozek T., Kirpotina L.N., Khlebnikov A.I., Quinn M.T. (2020). Chemical composition and immunomodulatory activity of hypericum perforatum essential oils. Biomolecules.

[17] Galeotti N. (2017). Hypericum perforatum (St John’s wort) beyond depression: A therapeutic perspective for pain conditions. J. Ethnopharmacol..

[18] Alahmad A., Feldhoff A., Bigall N.C., Rusch P., Scheper T., Walter J.G. (2021). *Hypericum perforatum* L.-mediated green synthesis of silver nanoparticles exhibiting antioxidant and anticancer activities. Nanomaterials.

[19] Złotek U. (2018). Antioxidative, potentially anti-inflammatory, and antidiabetic properties, as well as oxidative stability and acceptability of cakes supplemented with elicited basil. Food Chem..

[20] Jakubczyk A., Ćwiek P., Rybczyńska-Tkaczyk K., Gawlik-Dziki U., Złotek U. (2020). The influence of millet flour on antioxidant, anti-ACE, and anti-microbial activities of wheat wafers. Foods.

[21] Singleton V.L., Orthofer R., Lamuela-Raventós R.M. (1999). Oxidants and Antioxidants Part A.

[22] Lamaison J., Carnet A. (1990). Teneurs en principaux flavonoids des fleurs de Crataegeus monogyna Jacq et de Crataegeus laevigata (Poiret D. C) en fonction de la vegetation. Pharm. Acta Helv..

[23] Witkowska-Banaszczak E., Radzikowska D., Ratajczak K. (2018). Chemical profile and antioxidant activity of Trollius europaeus under the influence of feeding aphids. Open Life Sci..

[24] Miller G.L. (1959). Use of Dinitrosalicylic Acid Reagent for Determination of Reducing Sugar. Anal. Chem..

[25] Adler-Nissen J. (2002). Determination of the degree of hydrolysis of food protein hydrolysates by trinitrobenzenesulfonic acid. J. Agric. Food Chem..

[26] Khattab R.Y., Arntfield S.D. (2009). Functional properties of raw and processed canola meal. LWT Food Sci. Technol..

[27] Durak A., Baraniak B., Jakubczyk A., Świeca M. (2013). Biologically active peptides obtained by enzymatic hydrolysis of Adzuki bean seeds. Food Chem..

[28] Gawlik-Dziki U., Dziki D., Świeca M., Sȩczyk Ł., Rózyło R., Szymanowska U. (2015). Bread enriched with *Chenopodium quinoa* leaves powder—The procedures for assessing the fortification efficiency. LWT Food Sci. Technol..

[29] Re R., Pellegrini N., Proteggente A., Pannala A., Yang M., Rice-Evans C. (1999). Antioxidant Activity Applying an Improved Abts Radical Cation Decolorization Assay. Free Radic. Biol. Med..

[30] Brand-Williams W., Cuvelier M.E., Berset C. (1995). Use of a free radical method to evaluate antioxidant activity. Lebensm.-Wiss. Technol..

[31] Decker E.A., Welch B. (1990). Role of Ferritin as a Lipid Oxidation Catalyst in Muscle Food. J. Agric. Food Chem..

[32] Jakubczyk A., Karaś M., Złotek U., Szymanowska U., Baraniak B., Bochnak J. (2019). Peptides obtained from fermented faba bean seeds (*Vicia faba*) as potential inhibitors of an enzyme involved in the pathogenesis of metabolic syndrome. LWT Food Sci. Technol..

[33] Szymanowska U., Jakubczyk A., Baraniak B., Kur A. (2009). Characterisation of lipoxygenase from pea seeds (Pisum sativum var. Telephone L.). Food Chem..

[34] Jakubczyk A., Świeca M., Gawlik-Dziki U., Dziki D. (2018). Nutritional potential and inhibitory activity of bread fortified with green coffee beans against enzymes involved in metabolic syndrome pathogenesis. LWT Food Sci. Technol..

[35] Jakubczyk A., Kara M., Szychowski K.A., Binduga U.E., Dziedzic M., Zieli E., Rybczy K. (2020). Characterisation of Biologically Active Hydrolysates and Peptide Fractions of Vacuum Packaging String. Foods.

[36] Jakubczyk A., Złotek U., Szymanowska U., Krystyna J., Rybczy K. (2020). In Vitro Antioxidant, Anti-inflammatory, Anti-metabolic Syndrome, Antimicrobial, and Anticancer Effect of Phenolic Acids Isolated from Fresh Lovage Leaves [Levisticum officinale Koch] Elicited with Jasmonic Acid and Yeast Extract. Antioxidants.

[37] Bystroma L., Lewisa B., Brown D., Rodriguez E., Obendorfd R. (2008). Characterization of phenolics by LC-UV/vis, LC-MS/MS and sugars by GC in Melicoccus bijugatus Jacq. ‘Montgomery’ fruits. Food Chem..

[38] Dziki D., Cacak-Pietrzak G., Hassoon W., Gawlik-Dziki U., Sułek A., Różyło R., Sugier D. (2021). The fruits of sumac (Rhus coriari a L.) as a functional additive and salt replacement to wheat bread. LWT Food Sci. Technol..

[39] Lachowicz S., Świeca M., Pejcz E. (2021). Biological activity, phytochemical parameters, and potential bioaccessibility of wheat bread enriched with powder and microcapsules made from Saskatoon berry. Food Chem..

[40] Pycia K., Ivanišová E. (2020). Physicochemical and Antioxidant Properties of Wheat Bread Enriched with Hazelnuts and Walnuts. Foods.

[41] Biernacka B., Dziki D., Gawlik-Dziki U., Różyło R. (2020). Common wheat pasta enriched with cereal coffee: Quality and physical and functional properties. LWT Food Sci. Technol..

[42] Teterycz D., Sobota A. (2020). Legume flour as a natural colouring component in pasta production. J. Food Sci. Technol..

[43] Parizad P.A., Marengo M., Bonomi F., Scarafoni A. (2020). Bio-Functional and Structural Properties of Pasta Enriched with a Debranning Fraction from. Foods.

[44] Ranok A., Dissamal P., Kupradit C., Khongla C., Musika S., Mangkalanan S. (2021). Physicochemical properties and antioxidant activity of gluten-free riceberry-cheese cracker under simulated gastrointestinal transit. J. Food Sci. Technol..

[45] Assiri K., Alyami Y., Uyanik J.M., Romero-Reyes M. (2017). Hypericum perforatum (St. John’s Wort) as a possible therapeutic alternative for the management of trigeminal neuralgia (TN)—A case report. Complement. Ther. Med..

[46] Karaś M., Jakubczyk A., Szymanowska U., Złotek U., Zielińska E. (2017). Digestion and bioavailability of bioactive phytochemicals. Int. J. Food Sci. Technol..

[47] Köhn C.R., Fontoura A.M., Kempka A.P., Demiate I.M., Kubota E.H., Prestes R.C. (2015). Assessment of different methods for determining the capacity of water absorption of ingredients and additives used in the meat industry. Int. Food Res. J..

[48] Godswill C., Somtochukwu V., Kate C. (2019). The Functional Properties of Foods and Flours. Int. J. Adv. Acad. Res. Sci..

[49] Akubor P.I., Owuse A.U. (2020). Chemical Composition, functional and biscuit making properties of tomato peel flour. South Asian J. Food Technol. Environ..

[50] Farré R., Fiorani M., Rahiman S.A., Matteoli G. (2020). Intestinal permeability, inflammation and the role of nutrients. Nutrients.

[51] Karaś M., Jakubczyk A., Szymanowska U., Krystyna J., Lewicki S., Złotek U. (2019). Different temperature treatments of millet grains affect the biological activity of protein hydrolyzates. Nutrients.

[52] Jakubczyk A., Karaś M., Baraniak B., Pietrzak M. (2013). The impact of fermentation and in vitro digestion on formation angiotensin converting enzyme (ACE) inhibitory peptides from pea proteins. Food Chem..

[53] Świeca M., Gawlik-Dziki U., Dziki D., Baraniak B. (2017). Wheat bread enriched with green coffee—In vitro bioaccessibility and bioavailability of phenolics and antioxidant activity. Food Chem..

[54] Świeca M., Sȩczyk Ł., Gawlik-Dziki U., Dziki D. (2014). Bread enriched with quinoa leaves—The influence of protein-phenolics interactions on the nutritional and antioxidant quality. Food Chem..

[55] Wei L.H., Chen T.R., Fang H.B., Jin Q., Zhang S.J., Hou J., Yu Y., Dou T.Y., Cao Y.F., Guo W.Z. (2019). Natural constituents of St. John’s Wort inhibit the proteolytic activity of human thrombin. Int. J. Biol. Macromol..

[56] Béjaoui A., Salem I.B., Rokbeni N., M’rabet Y., Boussaid M., Boulila A. (2017). Bioactive compounds from Hypericum humifusum and Hypericum perfoliatum: Inhibition potential of polyphenols with acetylcholinesterase and key enzymes linked to type-2 diabetes. Pharm. Biol..

[57] Dekker I.P., Marijnissen R.M., Giltay E.J., Mast R.C.V.D., Oude R.C., Rhebergen D., Rius N. (2019). The role of metabolic syndrome in late-life depression over 6 years: The NESDO study. J. Affect. Disord..

[58] Bintsis T. (2017). Foodborne pathogens. AIMS Microbiol..

[59] Avato P., Raffo F., Guglielmi G., Vitali C., Rosato A. (2004). Extracts from St John’s Wort and their antiMicrobial Activity. Phyther. Res..

[60] Heydarian M., Jooyandeh H., Nasehi B., Noshad M. (2017). Characterization of Hypericum perforatum polysaccharides with antioxidant and antimicrobial activities: Optimization based statistical modeling. Int. J. Biol. Macromol..

[61] Lyles J.T., Kim A., Nelson K., Bullard-Roberts A.L., Hajdari A., Mustafa B., Quave C.L. (2017). The chemical and antibacterial evaluation of St. John’s Wort oil macerates used in Kosovar traditional medicine. Front. Microbiol..

[62] Süntar I., Oyardi O., Akkol E.K., Ozçelik B. (2016). Antimicrobial effect of the extracts from Hypericum perforatum against oral bacteria and biofilm formation. Pharm. Biol..

[63] Silva B.A., Malva J.O., Dias A.C.P. (2008). St. John’s Wort (*Hypericum perforatum*) extracts and isolated phenolic compounds are effective antioxidants in several in vitro models of oxidative stress. Food Chem..

