# Supplementary material for: The Influence of Hypericum perforatum L. Addition to Wheat Cookies on Their Antioxidant, Anti-Metabolic Syndrome, and Antimicrobial Properties
Source: Foods. 2021 Jun 15;10(6):1379. doi: 10.3390/foods10061379 (PMC8232325; doi:10.3390/foods10061379)
Supplement: Supplementary file 1 [file foods-10-01379-s001.zip › foods-1253531-SI.pdf]

Table S1. The parameters for all the molecules monitored by MRM method: precursor (Q1), product ions (Q2) as well as collision energy (CE) and retention times

No.	Compound	Precursor ion Q1 (m/z)	Product ion Q2 (m/z)	CE (V)	Retention time (min)
1.	Gallic acid	169.0	124.9	13	7.80
2.	Chlorogenic acid	352.8	191.0	14	8.34
3.	3,4-Didydroxybenzoic acid	152.9	108.0	20	8.36
4.	Daidzin	415.2	251.9	30	9.35
5.	Rutin	609.1	270.7	73	9.80
6.	Caffeic acid	178.8	135.0	12	9.90
7.	Syringic acid	197.0	182.0	10	10.37
8.	Naringin	579.1	271.1	30	12.06
9.	p-Coumaric acid	163.0	118.9	17	12.50
10.	Sinapinic acid	223.1	208.0	10	12.88
11.	Vanillin	151.0	135.8	10	13.00
12.	Ferulic acid	193.1	178.1	13	13.14
13.	m-Coumaric acid	163.0	118.9	17	13.50
14.	Rosmarinic acid	359.0	161.0	14	13.76
15.	Salicylic acid	136.8	93.1	10	16.33
16.	Quercetin	300.9	151.0	18	17.50
17.	trans-Cinnamic acid	147.0	102.8	10	18.00
18.	Naringenin	270.9	118.9	24	18.30
19.	2-Thiobarbituric acid (ISTD)	143.0	75.0	18	5.58

Table S2. Antimicrobial activity of tested samples towards bacteria and yeast; MIC – minimum inhibitory concentration, MLC - minimum lethal concentrations, ns – not studied.

**Antimicrobial activity**	**Sample**								
	**hydrolysates**			**fractions**			**controls**		
	**control**	**0.5 SJW**	**1.0 SJW**	**control**	**0.5 SJW**	**1.0 SJW**	**P[1]**	**S[2]**	**C[3]**
E. coli ATCC 25922	12.5*/>12.5**	12.5/>12.5	12.5/>12.5	12.5/>12.5	12.5/>12.5	12.5/>12.5	7.81/7.81	15.62/15.62	ns
S. aureus ATCC 29737			nd				>250/>250	7.81/7.81	ns
S. enterica ATCC 4931			nd				7.81/7.81	3.90/3.90	ns
B. cereus ATCC 14579	12.5/>12.5**	12.5/>12.5	12.5/>12.5	12.5/>12.5	12.5/>12.5	12.5/>12.5	>250/>250	15.62/15.62	ns
L. monocytogenes ATCC BAA-2660			nd				1.95/1.95	3.90/3.90	ns
C. albicans ATCC 90028							ns	ns	15.62/15.62

* Minimum inhibitory concentration MIC (mg mL^{-1}), ** Minimum lethal concentrations (MLC) (mg mL^{-1}), 1 – penicilin G, 2- streptomycine, 3- cyclohexamid, all antibiotics were used at concentrations μg mL^{-1}
